# Epstein-Barr virus: the molecular virology and the associated diseases

**DOI:** 10.20407/fmj.2022-018

**Published:** 2022-10-28

**Authors:** Takayuki Murata

**Affiliations:** Department of Virology and Parasitology, Fujita Health University, School of Medicine, Toyoake, Aichi, Japan

**Keywords:** EBV, Lymphoma, Carcinoma, Infectious mononucleosis, Autoimmune diseases

## Abstract

Ever since its discovery as the first human oncogenic virus, Epstein-Barr virus (EBV) has been the focus of many researchers and is one of the best-studied pathogens. EBV is a major causative agent of Burkitt lymphoma, Hodgkin lymphoma, post-transplantation lymphoproliferative disorder, NK/T cell lymphoma, chronic active EBV disease, nasopharyngeal carcinoma, gastric carcinoma, and infectious mononucleosis. Although a truly comprehensive understanding of the virus and the associated disorders remains elusive, major breakthroughs in molecular cloning and omics analyses are shedding new light on this important virus. For example, EBV is now implicated in autoimmune diseases and neurodegenerative disorders. This review provides an overview of the molecular biology of EBV, the research history, the associated disorders, and the epidemiology.

## Introduction

Epstein-Barr virus (EBV) is a human pathogen in the Herpesviridae family; a family that contains the α-, β-, and γ-herpesvirus subfamilies. The human α- and β-herpesviruses include herpes simplex virus, varicella zoster virus, and cytomegalovirus. EBV and Kaposi sarcoma-associated herpesvirus are members of the γ-herpesvirus subfamily and are both oncogenic. EBV infection of resting B cells leads to efficient B-cell transformation *in vitro*, reflecting the initial step of B-cell oncogenesis. Viral transmission between individuals most often occurs via saliva. The virus predominantly targets B cells, although it can also infect epithelial cells.^1,2^ Like other typical human herpesviruses, EBV is ubiquitous, asymptomatically infecting more than 90% of adults worldwide.

EBV infection during infancy is generally asymptomatic, though initial infection during or after adolescence can sometimes trigger infectious mononucleosis (IM). Because EBV can establish a latent infection and thus evade host immunity, the virus is impossible to eliminate, and most people will remain asymptomatic for life.^3,4^ However, EBV can cause low frequencies of several types of cancers: Burkitt lymphoma (BL), Hodgkin lymphoma (HL), post-transplantation lymphoproliferative disorder (PTLD), chronic active EBV disease (CAEBV), NK/T cell lymphoma (NKTCL), nasopharyngeal carcinoma (NPC), and gastric carcinoma (GC).^4,5^ In addition, EBV has been implicated in the development of some immunological diseases, including Sjogren’s syndrome (SS), systemic lupus erythematosus (SLE), rheumatoid arthritis (RA), multiple sclerosis (MS), and systemic sclerosis (SSc).^6–10^

Basic research on EBV has been challenging, as the cell tropism is narrow, the genome is large, there are repetitive sequences, and replication is inefficient. Clinical EBV research has been hampered by the low incidence of the associated disorders, regional differences, and the low ratio of EBV-positive cell in healthy individuals (only 1 in 10^5^ B cells are EBV-positive). Nevertheless, more than 37,000 EBV papers have been published as of 2022, and the number is increasing every year. EBV is one of the most extensively studied human viral pathogens; recent technological advances have greatly enhanced progress. In this review, we provide an overview of EBV and the associated disorders from molecular virological, historical, epidemiological, and clinical perspectives.

## Molecular biology of EBV

### Viral structure

EBV has a linear double-stranded DNA genome of approximately 175 kilobases, although the genome becomes circular in cells. The viral genome encodes more than 80 genes and 40 non-coding RNAs, and is incorporated in an icosahedral capsid. The nucleocapsid is enclosed within a lipid envelope (creating a virion); the space between the nucleocapsid and the envelope is termed the tegument.^4^

### Latent infection

Like other herpesviruses, EBV exhibits both latent and lytic states.^11,12^ During latency, EBV expresses only a limited number of genes, and the genome exists as an episome inside the nucleus. Latent infection is categorized into latency 0, I, IIa, IIb, and III types by the expression patterns of EBV genes^4,13^ (Table 1). Of the >80 open reading frames (ORFs), nine encode for proteins that are indisputably associated with latency: EBV nuclear antigen 1 (EBNA1), EBNA2, EBNA3A, EBNA3B, EBNA3C, EBNA leader protein (EBNA-LP), latent membrane protein 1 (LMP1), LMP2A, and LMP2B. Latency III status is evident in transformed B cells, commonly termed lymphoblastoid cell lines (LCLs), and in PTLD, all nine latent viral genes are expressed. In HL, CAEBV, NKTCL, and NPC, the production of EBNA2, EBNA3A, EBNA3B, EBNA3C, and EBNA-LP are restricted because the C and W promoters (Cp and Wp) are inactive. EBNA1 is expressed from the Q promoter (Qp), and LMP expression is preserved. The latency type IIb can be seen in a proportion of B cells in patients with IM, and in infected primary B cells for a few days or one week after infection. The infected B cells cannot express LMPs, but all EBNAs are expressed. Cancer cells from patients with BL and GC typically express EBNA1, but no other latent gene; this is characteristic of latency type I. The most silent pattern, latency type 0, in which no viral protein is detectable, can be found in EBV-positive memory B cells in asymptomatic healthy individuals.

If infected cells express more latent EBV genes (such as found in latency type III), the latent genes confer a growth advantage on the cells, but provide more antigens that may attract the attention of host immunity. Conversely, if the cells express limited numbers of latent genes (e.g., as in latency type I), the cells may evade the host immune system, but will require more genetic and/or epigenetic alterations for proliferation. In other words, the EBV latent gene expression pattern is determined at least partly by the balance between the individual or local host immune response and the ability of cells to proliferate.

Apart from these latent ORFs, other viral non-coding genes are expressed in latent cells, including the EBV-encoded RNAs (EBERs) and microRNAs (miRs) encoded in the *Bam*HI H fragment rightward frame 1 (BHRF1) and the *Bam*HI A fragment rightward transcript (BART) regions. EBERs are abundantly and ubiquitously transcribed by RNA polymerase III, even in latency type 0. miR-BARTs are more abundant in the latency types I and II of GC, NPC and NKTCL, whereas miR-BHRF1s are more highly expressed during type III latency.^14^

Some other genes are also expressed in latent cells. For example, the viral homolog of the *BCL-2* gene, BHRF1, is expressed (under the control of Wp) and contributes to B-cell transformation.^15–17^ The BNLF2A protein, that blocks antigen presentation by inhibiting TAP, is expressed during latency and protects infected cells from immune recognition.^18,19^

### Latent genes

#### EBNA1

The protein EBNA1 is not required to initiate B-cell transformation, but it is required for efficient, continued cell proliferation.^20^ EBNA1 tethers the EBV episome to the host chromosome via mechanical binding between the oriP motifs of the EBV genome and the chromosome. Such tethering is important for delivery of viral episomes into the nuclei of daughter cells. In addition, by binding to oriP, EBNA1 mediates latent viral DNA synthesis and activates the Cp and LMP1 promoter.^21^ As the presence of EBNA1 is sufficient for both replication and maintenance of a recombinant plasmid bearing oriP sequence in mammalian cells, the combination creates an efficient expression vector.

The EBNA1 protein is composed of N-terminal region (amino acid (aa) 1–89), a Gly-Ala repeat (GAr) (aa 90–326), and C-terminal region (aa 327–641). Given this very repetitive and lengthy sequence of GAr, EBNA1 translation is inhibited, and antigen presentation is reduced, aiding in the evasion of host immunity.^22^ EBNA1 also has a motif rich in Gly and Arg and is thus termed the GR motif. The GR motif is divided into two parts, with each part at either end of the GAr. The basic residues present in the GR domains allow EBNA1 to “stick” to the chromosome.^23^

Upon infection of B cells, EBNA1 is initially transcribed from the Wp and later from the Cp. In the latency types I and IIa, both Wp and Cp are inactive; however, EBNA1 expression is maintained because another latent promoter, Qp, is active. As Qp is activated in a broad range of cell types, EBNA1 can be found in all EBV-positive B, epithelial, and NK/T cancer cells.^13,24^

#### EBNA2

The protein EBNA2 is a transcriptional cofactor transcribed from Wp immediately after infection of B cells, and is later transcribed from Cp.^21,25^ While EBNA2 does not directly bind DNA, this protein can either induce or suppress transcription of cellular and viral genes by associating with cellular transcription factors such as RBPJκ, PU.1, EBF, NF-κB, and RUNX1.^26–29^ Additionally, EBNA2 is a component of several super-enhancers, as are EBNA-LP and other viral and cellular transcriptional activators.^30,31^ EBNA2 induces transcription of many cellular genes, of which one of the most important is the MYC,^32,33^ a transcription factor with basic helix-loop-helix and leucine zipper motifs. MYC up-regulates and down-regulates cellular genes involved in the cell cycle, apoptosis, and nucleotide metabolism, and potently promotes cell proliferation. Indeed, MYC upregulation has been reported in many types of cancers and is associated principally with B-cell oncogenesis. EBNA2 also activates the viral LMP1 promoter and Cp.^34,35^ EBNA2 is not expressed in epithelial or NK/T cells.^13^

#### EBNA-LP

EBNA-LP transcription commences at the Wp and Cp in latency type III, like EBNA2. Most of the protein is encoded by the W repeat (also termed the IR1) region, and therefore the sequence is very repetitive. EBNA-LP associates with the viral transcriptional cofactor EBNA2 to increase transcriptional activity.^36^ Presence of EBNA-LP can promote the transformation of adult B cell, but is not required for this process; in contrast, EBNA-LP is essential for transformation of naïve B cells.^37^

#### EBNA3A/B/C

The EBNA3 cluster has three members: A, B, and C. The sequences have similarity; the genes lie adjacent in the viral genome and are transcribed from the same promoters (Wp and Cp). EBNA3 proteins have RBPJ-binding domains in the N-terminal regions and thus function as regulatory cofactors of EBNA2.^38^ EBNA3A and EBNA3C play critical roles in the efficient transformation of B cells. For example, EBNA3A and 3C repress the transcription of p14^ARF^ and p16^INK4A^ (that are CDK inhibitors that block cell cycling), thereby promoting the proliferation of infected B cells.^39^ Conversely, EBNA3B is not only not required for B-cell transformation; it is in fact a tumor suppressor.^40^ Notably, the sequences of the EBNA3s differ between EBV-1 and EBV-2.^41^

#### LMP1

The LMP1 gene is transcribed from a unique promoter in the latency types IIa and III.^42,43^ Although expression of the gene is limited to resting B cells for several days after infection,^25^ expression is later increased by EBNA2 protein.^44^ LMP1 is regarded as a oncoprotein and is required for the efficient transformation of B cells. LMP1 is a transmembrane protein with six transmembrane domains and two intracellular effector domains termed C-terminal activation regions 1 and 2 (CTAR1 and 2) or transformation effector sites 1 and 2 (TES1 and 2).^45^ The effector domains mediate constitutive activation of TNFR/CD40 signaling, activating the NF-κB, JNK, and MAPK pathways. LMP1 increases the expression of many genes in infected cells, including ICAM-1, LFA-1, BCL-2, and A20.^46–48^

#### LMP2A/B

LMP2A and B share a C-terminal sequence, but the N-terminal domains differ. The LMP2A gene has a unique promoter, but the LMP2B promoter shares *cis*-acting elements with the LMP1 promoter.^49^ Both genes are adjacent to each other and are expressed in latency types IIa and III, as is LMP1. LMP2A features 12 transmembrane domains in the C-terminus; the N-terminal region of the protein lies in the cytoplasm. This N-terminal region contains immunoreceptor tyrosine-based activation motifs and PPPY motifs, which mediate downstream signaling. LMP2A mimics the B-cell receptor (BCR) and constitutively activates signaling of Syk and PI3K/AKT.^50^ Knockout of LMP2A significantly reduces B-cell transformation by EBV, but the gene is not required for transformation *in vitro*.^17,51^

As LMP2B lacks the N-terminal cytoplasmic domain of LMP2A, LMP2B cannot elicit signaling. Rather, LMP2B serves as a negative regulator of LMP2A.^52^ The LMP2B gene is assumed to be non-essential for B-cell transformation. Studies on deletion mutants of the C-terminal transmembrane domains shared by LMP2A and B showed that neither gene was required for B-cell transformation if they were simultaneously disrupted,^53,54^ although deletion of LMP2A alone significantly reduced the transformation efficiency.^17,55^ Recent genomic analyses revealed that many B-cell lymphomas exhibit simultaneous deletions of LMP2A and LMP2B,^56^ also suggesting that LMP2A is oncogenic and that LMP2B is an LMP2A antagonist.

#### Lytic infection

Although the trigger(s) of EBV reactivation *in vivo* remain unclear, reactivation can be induced at the cell culture level by anti-immunoglobulin (Ig), TGF-β, TPA, an HDAC inhibitor, and hypoxia, that triggers rapid expression of lytic viral genes, extensive viral DNA synthesis in the nucleus, and progeny virion production.^57^ Lytic genes are divided into three classes by the timing of expression: Immediate-Early (IE), Early, and Late. Typical lytic genes are described in the following section.

### Lytic genes

#### BZLF1/BRLF1

After induction of reactivation, EBV rapidly expresses two IE genes, BZLF1 and BRLF1. These are both transcriptional activators that induce transcription of viral genes, especially Early genes. The BZLF1 gene encodes a b-ZIP-type transcription factor that has a unique ability to efficiently induce activation of CpG-methylated promoters.^58^ The BRLF1 protein induces transcription by either directly or indirectly binding to target gene promoters, or by activating cell signaling pathways.

#### Replication genes

Viral genes involved in synthesis of viral DNA are expressed during the Early phase. EBV encodes BALF5 (the catalytic polymerase subunit), BMRF1 (a polymerase processivity factor, also termed EA-D), BALF2 (a single-stranded-DNA-binding protein), BBLF4 (a helicase), BSLF1 (a primase), and BBLF2/3 (a primase-binding protein). Additionally, the BZLF1 protein serves as an origin-binding protein during lytic replication. The expression of these seven genes are required for viral DNA synthesis, as is the gene encoding BKRF3 (an uracil-DNA glycosylase).^59^

#### vPIC

Other Early proteins of the lytic stage include those that create the viral pre-initiation complex (vPIC), which induces transcription of Late viral genes after viral DNA synthesis.^60^ The vPIC includes BcRF1, BDLF3.5, BDLF4, BFRF2, BGLF3, and BVLF1. BcRF1 may be a TATT-binding protein; however, the roles of the other proteins remain unclear.

#### Structural proteins

Genes associated with the Late stage include many structural genes that encode for the capsid, tegument, and glycoproteins.^61^ Capsid proteins form the icosahedral container for the viral progeny genomic DNA. Tegument proteins are incorporated into the space between the nucleocapsid and envelope and play roles in progeny virion maturation, transportation, and enhancement of infectivity. Envelope glycoproteins are required for progeny virion maturation, transportation, viral attachment and cell entry.

## Disorders

### IM

EBV was associated with IM only a few years after the virus was discovered in BL.^62^ The symptoms of IM include fever, pharyngeal inflammation, lymphadenopathy, fatigue, and hepatosplenomegaly that persist for 1–2 weeks. Most IM patients exhibit leukocytosis and atypical lymphocytes. Many of these IM symptoms are attributable to the abnormal expansion of T cells that counter the initial EBV infection. EBV of IM patients exhibit expression patterns characteristic of either latency types IIb, III, or the lytic phase. Antibodies against lytic EBV genes (such as VCA and EA-D) can be detected in IM patients.

### BL

endemic BL (eBL), a disease endemic in sub-Saharan Africa, was first identified by Denis Burkitt in children.^63^ Michael Epstein, Bert Achong, and Yvonne Barr cultured cells from biopsied BL tissue from African patients and, in 1964, reported microscopic objects reminiscent of herpesvirus particles^64^; these particles were later recognized as EBV. The eBL is more frequent in men and children than women; the tumor typically grows rapidly in an area between the upper jaw and the abdomen. Almost 100% of eBL is associated with EBV, but the association is lower for sporadic BL (sBL). Tumors of both eBL and sBL patients characteristically have translocations between the MYC and IgH (or IgL) genes; the oncogenic MYC gene is thus overexpressed in BL cells.^65^

EBV of BL cells usually exhibits the latency type I gene expression pattern. However, after long-term *in vitro* culture, there can be a change to latency type III status, whereby all latent genes are expressed. The expression of EBNA and LMP is limited *in vivo*, which enables the virus to avoid the host immune system; however, in the absence of an immune response (e.g. *in vitro*) expression of EBNA and LMP may confer a growth advantage to the cells. In addition to the MYC translocation, BL often features somatic mutations in TP53, ID3, RET, SWI/SNF, and ARID1A.^66^

### HL

HL is a B-cell lymphoma identified by Thomas Hodgkin in 1832. Histologically, the essential feature of the disorder is that of large tumor cells, termed Hodgkin and Reed–Sternberg (HRS) cells, surrounded by non-malignant inflammatory cells including T cells, macrophages, and fibroblasts. As HRS cells cannot grow when isolated, the surrounding cells are presumed to support HRS proliferation. EBV was linked to HL as early as 1969, when HRS-like cells were found in IM patients.^67^ Additionally, a history of IM was associated with a higher risk of developing HL.^68^ However, a direct link between EBV and HL was not found until 1987, when EBV DNA was detected in HL tissues.^69^ EBV positivity is dependent on the lymphoma subtype; EBV is present in approximately 70% of mixed cellularity HL, >95% of lymphocyte-depletion HL, 10–40% of nodular sclerosis, and 0% of lymphocyte-predominant HL.^70^ The incidence of HL is higher in the West, accounting for about 30% of all lymphomas, but is lower in the East, such as in Japan (≤5%). EBV of HL typically exists in the latency type II pattern, suggesting the involvement of LMP1 and LMP2A in oncogenesis. Furthermore, EBV-positive HRS cells sometimes lack functional BCR (reflecting apoptotic cell death in the germinal center) but LMP1 and LMP2A may inhibit apoptosis and thus compensate for the absence of BCR.^71–73^ Somatic mutations in genes encoding NF-κB signaling molecules have also been reported.^74^

### PTLD and AIDS-related lymphoproliferative disease

In patients with congenital or acquired immunodeficiency, or who are prescribed immunosuppressants, lymphoproliferative disease (LPD) or lymphomas are common. EBV was first linked to PTLD in 1980^75^; it is now known that 60% to 90% of PTLD cases are associated with EBV.^76^ The terms “PTLD” and “AIDS-related LPD” are generic in nature; they in fact include heterogenous disorders. In the early stages, LPDs are often polyclonal or oligoclonal; later, the disease may appear similar to BL, HL, and diffuse large B-cell lymphoma (DLBCL). As the patient’s immunity is compromised, the EBV of LPDs expresses many latent genes, thus exhibiting latency type III status, but cases with restricted latent gene expression have also been described. MYC translocations and mutations in TP53 and BCL6 have been found in some PTLD cases,^77^ but such somatic mutations in EBV-positive PTLD patients are less frequent than in EBV-negative PTLD cases.^76^

### CAEBV and NKTCL

CAEBV and NKTCL are LPD and lymphoma of T or NK cells associated with EBV. The incidence is highest in east Asia; however, the reason for this is unknown.^78,79^ CAEBV is commonly found in subjects aged <20 years, while NKTCL is more frequently diagnosed in middle-aged or older men. EBV was first detected in patients with CAEBV and NKTCL in 1988^80^ and 1990,^81^ respectively. In both cases, the virus exhibits latency type II status, suggesting that LMP1 is important in terms of oncogenesis.^82^ Somatic mutation accumulations are found in DDX3X, KMT2D, TP53, BCOR, TET2, STAT3, ARID1A, and PD-L1.^56,83^ Furthermore, intragenic deletions of viral genes are frequently found in CAEBV and NKTCL specimens. Because these deletions can lead to more efficient expression of viral lytic genes, it is suggested that viral lytic cycle genes contribute to the pathogenesis of these disorders.^56^

### NPC

Even before Denis Burkitt reported eBL in Africa, the incidence of NPC in southern China was known to be higher than elsewhere in the world.^84–86^ Middle-aged or older males are at higher risk than other groups. Two years after the initial report of EBV in BL cells, EBV was first implicated in NPC.^87^ Sera from African and American NPC patients reacted with BL cell lines more strongly than sera from BL patients. Soon afterwards, EBV DNA was detected in NPC biopsy material.^88,89^ An oncogenic role for EBV in NPC was described in 2010; EBV reproducibly immortalized pre-malignant, nasopharyngeal epithelial cells.^90^ EBV infection triggered anchorage-independent cell growth, invasion, and survival even in the absence of growth factors or nutrients. The EBV present in NPC cells typically exhibit a latency type II pattern, suggesting that LMP1 plays a role in the oncogenesis of this type of cancer.^91^ Somatic mutations have been reported in CDNK2A, CCND1, ARID1A, AKT2, TP53, KRAS, and PIK3CA.^92^ Genomic analyses of the EBV in NPC specimens revealed that certain nucleotide variations, especially in the BALF2 gene, are highly associated with an increased risk of NPC, in the region-dependent manner^93,94^; this suggests the contribution of viral lytic cycle in the carcinogenesis.

### GC

The presence of EBV in GC was first reported in 1992^95^; it is now known that approximately 10% of GCs are EBV-positive. In patients with EBV-positive GC, both the host and viral genomes exhibit more extensive CpG methylation than in patients with EBV-negative GC.^96^ In addition to epigenetic alterations, somatic mutations in TP53, KRAS, ARID1A, PIK3CA, BCOR, and PD-L1 are present in GC cells.^97^ The EBV of GCs generally exhibits restricted expression of latent genes (latency type I), but involvement of LMP2A has also been suggested.^98^ Histologically, infiltrations of lymphocytes are typically found in EBV-positive epithelial cancers, GC, and NPC.

### SS/SLE/RA/MS/SSc

SS, SLE, RA, MS, and SSc are complex autoimmune diseases. Both genetic and environmental factors play crucial roles; EBV has been suggested to be an environmental risk factor for the development of these diseases.^8^ One of the earliest links discovered between EBV and autoimmune disease was that some patients with IM developed SS or SLE. Additional circumstantial evidence followed; the levels of EBV antigen, antibody, and DNA were higher than normal in either the peripheral blood or the disease loci of SS and SLE patients. Recently, a study in the United States of 10 million young adults revealed that the MS incidence increased 32-fold after *de novo* EBV infection but not after other infections.^6^

Several pathogenetic mechanisms have been proposed: (i) an EBV antigen may mimic a host autoantigen; (ii) EBV infection may induce an excessive or abnormal immune response, destroying self-tolerance; and/or, (iii) salivary gland cells may be directly killed by EBV lytic reactivation. The GAr of EBNA1 can mimic the autoantigens of RA and MS.^99,100^ Single-cell repertoire analysis of the B cells of MS patients revealed molecular mimicry between a non-GAr epitope of EBNA1 and a glial cell adhesion molecule expressed in the central nervous system.^101^ The viral transcriptional cofactor EBNA2 can trigger dysregulation of host autoimmunity risk loci.^102,103^ LMP1 and LMP2A constitutively transmit CD40 and BCR signals, respectively, facilitating the survival of auto-reactive B cells in the germinal center.^104,105^ One viral lytic gene, vIL-10 (BCRF1), can alter the balance of the immune system.^9^ A synergistic effect between a patient’s genomic predisposition and an environmental factor has also been suggested; genome-wide association studies found that HLA-DR15 was associated with the strongest predisposition toward MS, and EBV is amplified more efficiently (because of weaker anti-EBV immunity) in patients with the HLA-DR15 allele.^10^

## Conclusion

Recent technological advances in genetic and epigenetic analyses have enhanced our molecular understanding of EBV and have revealed further features of disorders caused by the virus. We propose that our next goal should therefore be the development of effective preventative and therapeutic measures. Such work is already in progress worldwide.

## Figures and Tables

**Table1:** EBV latent genes and their expression patterns

Latent gene	Function	Necessary for B cell transformation	Latency type 0	Latency type I	Latency type II (or IIa)	Latency type IIb	Latency type III
			Memory B	BL, GC	HL, CAEBV, NKTCL, NPC	IM, pre-latency	IM, PTLD, LCL
EBERs	Non-coding RNA	No	+	+	+	+	+
EBNA1	Tethers episomal DNA to chromosome	Essential		+	+	+	+
EBNA2	Transcriptional cofactor	Essential				+	+
EBNA-LP	Cofactor of EBNA2	Essential for naïve B, important for memory B				+	+
EBNA3A	Cofactor of EBNA2	Essential				+	+
EBNA3B	Cofactor of EBNA2	No (tumor suppressor)				+	+
EBNA3C	Cofactor of EBNA2	Essential				+	+
LMP1	CD40 mimic, activates NFkB pathway	Essential			+		+
LMP2A	BCR mimic, activates AKT pathway	Important			+		+
LMP2B	Negative regulator of LMP2A	No			+		+

BCR, B cell receptor; BL, Burkitt lymphoma; CAEBV, chronic active Epstein-Barr virus; EBERs, Epstein-Barr virus-encoded small RNAs; EBNA1, Epstein-Barr virus nuclear antigen 1; EBNA2, Epstein-Barr virus nuclear antigen 2; EBNA3A, Epstein-Barr virus nuclear antigen 3A; EBNA3B, Epstein-Barr virus nuclear antigen 3B; EBNA3C, Epstein-Barr virus nuclear antigen 3C; EBNA-LP, Epstein-Barr virus nuclear antigen leader protein; EBV, Epstein-Barr virus; GC, gastric carcinoma; HL, Hodgkin lymphoma; IM, infectious mononcleosis; LCL, lymphoblastoid cell lines; LMP1, latent membrane protein 1; LMP2A, latent membrane protein 2A; LMP2B, latent membrane protein 2B; NKTCL, NK/T-cell lymphoma; NPC, nasopharyngeal carcinoma; PTLD, post-transplant lymphoproliferative disease.
